# BAD Modulates Counterregulatory Responses to Hypoglycemia and Protective Glucoprivic Feeding

**DOI:** 10.1371/journal.pone.0028016

**Published:** 2011-12-05

**Authors:** Mayowa A. Osundiji, Marina L. Godes, Mark L. Evans, Nika N. Danial

**Affiliations:** 1 Department of Cancer Biology, Dana-Farber Cancer Institute, Boston, Massachusetts, United States of America; 2 Department of Cell Biology, Harvard Medical School, Boston, Massachusetts, United States of America; 3 University of Cambridge Metabolic Research Laboratories, Department of Medicine and National Institute for Health Research Cambridge Biomedical Research Centre, Cambridge, United Kingdom; Boston University, United States of America

## Abstract

Hypoglycemia or glucoprivation triggers protective hormonal counterregulatory and feeding responses to aid the restoration of normoglycemia. Increasing evidence suggests pertinent roles for the brain in sensing glucoprivation and mediating counterregulation, however, the precise nature of the metabolic signals and molecular mediators linking central glucose sensing to effector functions are not fully understood. Here, we demonstrate that protective hormonal and feeding responses to hypoglycemia are regulated by BAD, a BCL-2 family protein with dual functions in apoptosis and metabolism. BAD-deficient mice display impaired glycemic and hormonal counterregulatory responses to systemic glucoprivation induced by 2-deoxy-D-glucose. BAD is also required for proper counterregulatory responses to insulin-induced hypoglycemia as evident from significantly higher glucose infusion rates and lower plasma epinephrine levels during hyperinsulinemic hypoglycemic clamps. Importantly, RNA interference-mediated acute knockdown of *Bad* in the brain provided independent genetic evidence for its relevance in central glucose sensing and proper neurohumoral responses to glucoprivation. Moreover, BAD deficiency is associated with impaired glucoprivic feeding, suggesting that its role in adaptive responses to hypoglycemia extends beyond hormonal responses to regulation of feeding behavior. Together, these data indicate a previously unappreciated role for BAD in the control of central glucose sensing.

## Introduction

Intensified lowering of blood glucose reduces the risk of chronic complications of type 1 diabetes; however, clinical attempts to achieve these benefits are limited by an increased risk of hypoglycemia induced by aggressive insulin therapy, also known as iatrogenic hypoglycemia [1], [2]. Blood glucose levels are normally maintained within narrow boundaries. Falling blood glucose levels are rapidly sensed and a myriad of counterregulatory responses are triggered to help limit hypoglycemia and aid restoration of normoglycemia. These protective responses include physiological neurohumoral changes such as increased release of glucagon, epinephrine and corticosterone, which act to increase endogenous glucose production, limit tissue glucose utilization, and trigger symptomatic responses, particularly hunger [1], [3]. Counterregulatory responses become impaired in some diabetic patients leading to further susceptibility to hypoglycemic episodes [3], [4].

Increasing evidence suggests that the brain plays a predominant role in sensing hypoglycemia or glucoprivation and initiating a series of adaptive responses leading to the release of counterregulatory hormones and protective glucoprivic feeding [5], [6], [7], [8], [9], [10], [11], [12]. The precise mechanisms utilized by the brain to detect glucose deficit and initiate counterregulatory responses are under active investigation. Several putative frameworks for the metabolic basis of central gluco-detection and the relevant metabolic signals/messengers have been put forward [13]. Multiple neuroanatomical areas, including paraventricular and ventromedial hypothalamus, nucleus of the solitary tract, dorsal motor nucleus of the vagus and basolateral medulla and several neural cell types play a pivotal role in this process [5], [6], [7], [8], [9], [10], [11], [12]. Genetic and pharmacologic approaches have identified several molecular mediators of central glucose sensing and counterregulation, including glucose transporter 2 (GLUT2) [14], [15], ATP-dependent K^+^ (K_ATP_) channels [16], [17], [18], glucokinase (GK) [19], [20], [21], [22], [23] and AMP-activated protein kinase (AMPK) [24], [25]. In addition, neurotransmitters such as γ-amino butyric acid (GABA) [26], [27], [28], [29], glutamate [30], N-methyl D-aspartate (NMDA) [31], as well as corticotrophin-releasing factor receptors [32] have been implicated in hypoglycemia counterregulation. However, the precise neurochemical nature of these responses and the coordinated function of the central glucose sensing pathways are not completely defined.

The BCL-2 family of cell death regulators constitutes a critical control point in the regulation of apoptosis [33], [34]. Although BCL-2 proteins are best known for their control of apoptosis, select proteins in this family carry physiologic roles or “day jobs” separate from apoptosis, including nutrient metabolism [35], [36], [37]. We have previously reported a novel role for the pro-apoptotic BCL-2 protein BAD (Bcl-2-associated death promoter) in islet β-cell glucose sensing through its association and activation of GK [37]. In the brain, BAD is present in several anatomical locations, including areas that are pivotal for brain nutrient sensing and control of glucose counterregulation [38]. Using a combination of genetic tools, including BAD-deficient mice and RNA interference approaches, we investigated the role of BAD in the control of counterregulatory hormonal and feeding responses to glucoprivation. Our results suggest that BAD's function in the brain is required for proper hypoglycemia detection and initiation of hormonal and feeding responses to glucoprivation.

## Materials and Methods

### Ethics Statement

All animal procedures were reviewed and approved by the Institutional Animal Care and Use Committees of Dana-Farber Cancer Institute and Vanderbilt University School of Medicine. Animal protocols 05–052 and 09–072 were used for these studies.

### Animals

The *Bad* ^−/−^ mice [39] were bred into the C57BL/6J genetic background for at least 14 generations and were validated by genome scanning to be 99.9% congenic with C57BL/6J. Wild type C56BL/6J mice used for brain knockdown experiments were purchased from the Jackson Laboratory (Bar Harbor, ME). Mice were housed in pathogen-free facility and maintained on a 12 h light/dark cycle.

### Glycemic Response to Glucoprivation

We studied 10 week old male *Bad* ^−/−^ and *Bad ^+/+^* mice (weight: 25.7±0.6 vs 25.1±0.5 g, respectively). Non-fasted *Bad* ^−/−^ and *Bad ^+/+^* mice had food taken away 30 min prior to i.p. injection of 150 mg/kg 2DG. Blood glucose levels were then monitored at 15 min intervals over the ensuing 60 minutes from tail vein blood using an automated glucose monitor (OneTouch Ultra, LifeScan).

### Hormonal Response to Glucoprivation

Non-fasted *Bad* ^−/−^ and *Bad* ^+/+^ mice had food taken away immediately prior to i.p. injection of 150 mg/kg 2DG or saline. Blood collection and hormone monitoring were carried out as described below.

### L-Arginine Stimulation Studies

Overnight fasted *Bad* ^−/−^ and *Bad ^+/+^* mice received i.p. injection of 3 g/kg L-arginine. Mice were anesthetized after 10 min by a brief exposure to isoflurane as described below and blood samples were collected via retro-orbital sinus puncture for glucagon measurements.

### Blood Collection for Glucose and Hormonal Assays in Glucoprivation and L-Arginine Stimulation Studies

**Figure 1:**
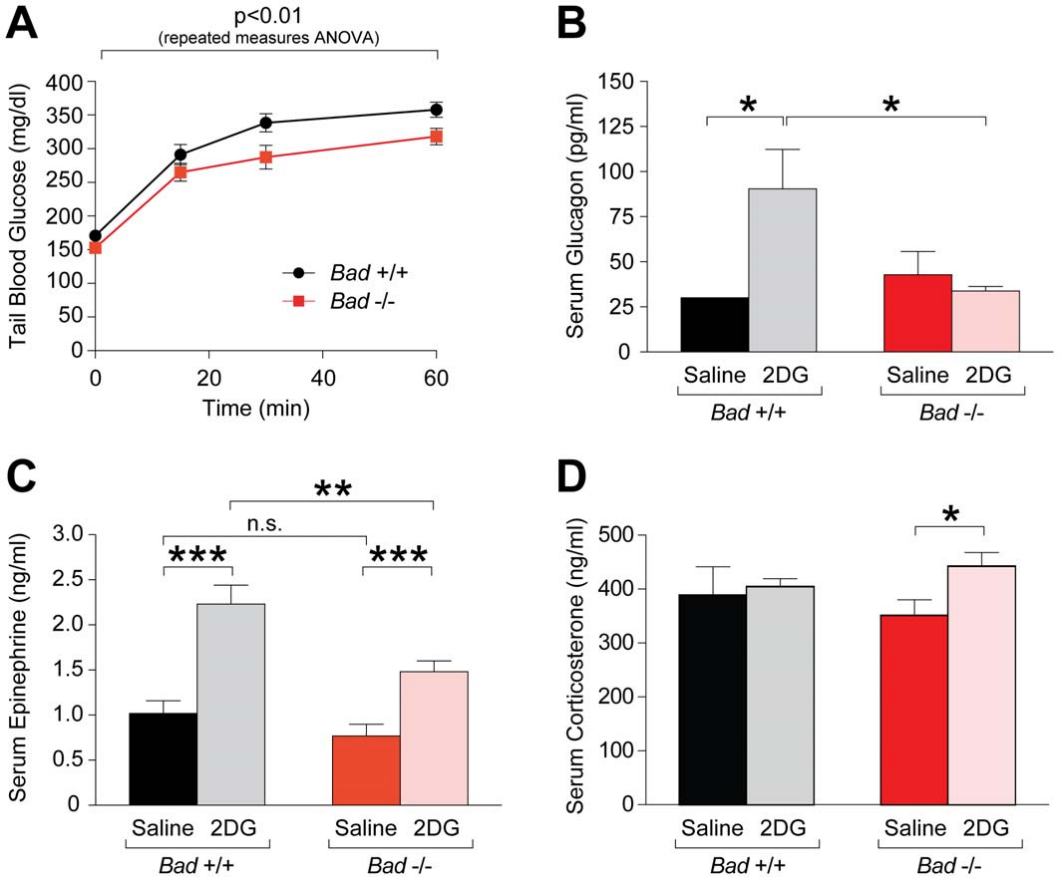
Impaired glycemic and hormonal counterregulatory responses to glucoprivation in BAD-deficient mice. (A) Glycemic response to systemic glucoprivation in *Bad* ^−/−^ and *Bad* ^+/+^ mice. Tail blood glucose was monitored over a 60 min period following intraperitoneal (i.p.) administration of 2DG (150 mg/kg) to *Bad* ^−/−^ (n = 12) and *Bad* ^+/+^ (n = 14) mice. p<0.01; *Bad* ^−/−^ vs *Bad* ^+/+^, two-way repeated measures ANOVA. (B–D) Hormonal responses to systemic glucoprivation. Serum glucagon (B), epinephrine (C), and corticosterone (D) levels were measured in retro-orbital blood samples collected 30 min after i.p. administration of 2DG (150 mg/kg) or saline to *Bad* ^−/−^ and *Bad* ^+/+^ mice (n = 7–17 per group). *p<0.05; **p<0.01; ***p<0.001, unpaired two tailed *t*-test.

Blood samples were collected using tail bleeds when blood glucose was continuously measured and small blood volume was required (as in Fig. 1A). Retro-orbital collection was used when relatively larger blood volume was needed for measurement of multiple serum hormones and blood glucose levels at one time point only. Briefly, following anesthetic induction with isoflurane (up to 5% in oxygen), blood samples were rapidly collected in the presence of 1 µg/ml aprotinin and 1 mM EDTA using retro-orbital puncture. Anesthesia was only maintained at the beginning of blood collection and retro-orbital puncture and blood sampling lasted ∼120 seconds. Serum samples were prepared and glucagon levels determined by RIA (Linco), epinephrine and corticosterone levels were determined by ELISA.

### Glucagon Secretion Studies in Primary Islet Cultures

*Bad* ^−/−^ and *Bad ^+/+^* primary islets were isolated by the collagenase method as previously described [37]. Glucagon was measured using a slight modification of a previously described method [18]. Briefly, freshly isolated islets were preincubated at 37°C for 60 min in Krebs–Ringer bicarbonate buffer, pH 7.4, supplemented with 20 mM HEPES, 0.05% bovine serum albumin and 7 mM glucose. The islets were then transferred to 500 µl of the Krebs–Ringer bicarbonate buffer containing 7 mM glucose and 50 µM carbachol, 16.7 mM glucose or 1 mM glucose in the presence of 500 KIU/ml of aprotinin and incubated for 60 min. Aliquots of the medium were then removed for glucagon measurements. For each condition, 15 tubes containing 10 islets/tube were assessed. Data shown are cumulative of 3 independent islet isolations.

### Hyperinsulinemic Hypoglycemic Clamp Studies

Hypoglycemic clamps were performed as previously described [40] using 12 week old male *Bad* ^−/−^ and *Bad ^+/+^* mice (weight: 25.7±1.1 vs 24.2±1 g, respectively, p = non-significant). Briefly, five days prior to the clamp study, catheters were inserted into the carotid artery and jugular vein. Chronically catheterized conscious mice were subjected to clamps under conscious and unstressed conditions after 6 hr fasting. A 1 µCi bolus of [3-^3^H] glucose was given at *t* = −120 min before insulin infusion, followed by a 0.05 µCi/min infusion for the duration of the study. After obtaining a baseline plasma glucose concentration (t = −0 min), a continuous infusion of insulin was initiated (10 mŨk
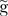
min) into the jugular vein. Plasma glucose levels were monitored every 10 min, and glucose was infused into the jugular vein at a variable rate to reach the target plasma glucose concentration (50 mg/dl). Mice received a continuous infusion of heparinized saline-washed erythrocytes into the jugular vein from donor animals to prevent a fall of hematocrit. Blood samples were also collected from the carotid artery for measurement of [3-^3^H] glucose, insulin (RIA; Millipore, Billerica, MA) and counterregulatory hormones including glucagon (RIA; Millipore, Billerica, MA), epinephrine, norepinephrine (HPLC; [41]), and corticosterone (RIA; MP Biomedicals, Orangeburg NY). Briefly, after deproteinization of plasma with barium hydroxide [Ba(OH)_2_, 0.3 N] and zinc sulfate [ZnSO_4_, 0.3 N], plasma [3-^3^H] glucose extracts were dried and radioactivity was determined by liquid scintillation counting (Packard TRI-CARB 2900TR) with Ultima Gold (Packard) as scintillant. Glucose appearance (*R*_a_) and disappearance (*R*_d_) were determined using Steele nonsteady-state equations. Endogenous glucose production (endo*R*_a_) was determined by subtracting the glucose infusion rate (GIR) from total *R*_a_ [42].

### Adenovirus Production

Adenoviruses carrying *Bad* shRNA or scrambled control were constructed using the Adeno-X Expression Systems 2 with Creator Technology (Clonetech). Briefly, 19 bp sequences based on the *Bad* coding sequence were designed using Block-it RNAi designer software (Invitrogen). Oligonucleotides were then designed according to the knockout clone and confirm PCR kit (Clontech) and cloned into RNAi-Ready pSIERN-DNR-Ds Red-Express Donor vector. Virus amplification, purification, titration and verification were carried out using the services of ViraQuest Inc. (North Liberty, IA).

### RNA-Interference Mediated Knockdown of *Bad* in the Brain

9 week old male C57BL/6J mice underwent stereotactic surgery for implantation of an indwelling bilateral guide cannula into the brain according to the following stereotactic coordinates: 1.3 mm posterior to bregma, ±0.5 mm from midline. The goal was to create whole brain knockdown of *Bad*. The infusion coordinates where targeting medial hypothalamus, immediately neighboring the third ventricle, which will facilitate whole brain re-distribution. After recovery, mice received brain infusion of adenoviruses containing *Bad* shRNA or scrambled control at a dose of 2×10^7^ plaque forming units/mouse. We selected an infusion volume (1 µl/mouse) to allow whole brain distribution of infusate. We validated the efficiency of brain knockdown by monitoring *Bad* mRNA levels in the brainstem and frontal cortex as representative hindbrain and forebrain areas, respectively. Seven days after virus delivery, mice were subjected to glucoprivation as described above.

### RNA Analysis

RNA was prepared from brain samples and quantitative PCR for *Bad* expression was performed as previously described [37]. Primers for cyclophilin served as internal controls for the quality of RNA. Primer sequences are available upon request.

### Glucoprivic Feeding Studies

We studied 11 week old male *Bad* ^−/−^ and *Bad ^+/+^* mice (weight: 26.4±0.4 vs 25.5±0.4 g, respectively, p = non-significant). Glucoprivic feeding experiments were conducted between 10 AM and 5 PM. *Ad libitum*-fed *Bad* ^−/−^ and *Bad ^+/+^* mice received i.p. injection of 150 mg/kg 2DG or saline. Food intake over the ensuring 4 hours was monitored by weighing the food remaining in the cages [19]. Paper towel bedding was used during glucoprivic feeding tests to aid the determination of in-cage food waste.

### Statistics

Data are presented as means ± s.e.m. Glucose excursion curve during glucoprivation studies in *Bad* ^−/−^ and *Bad* ^+/+^ mice (Fig. 1A) were analyzed by repeated measures ANOVA. Statistical analysis for all other experiments was performed using two tailed Student's *t*-test.

## Results

### Impaired Glycemic and Hormonal Responses to Glucoprivation in BAD-Deficient Mice

The effect of BAD on glucose sensing in β-cells [37] prompted investigation of its role in neuroendocrine glucose sensing and adaptive responses to hypoglycemia. Initial analysis of tail blood glucose concentration in response to a glucoprivic signal induced by intraperitoneal (i.p.) administration of 150 mg/kg 2-deoxy glucose (2DG) indicated small but significant differences with lower values in *Bad* ^−/−^ mice compared with *Bad ^+/+^* counterparts (Fig. 1A). The rise in blood glucose in response to glucoprivation stems from a counterregulatory neurohumoral response, which acts to increase endogenous glucose production and reduce peripheral glucose uptake [9]. Our study design in these experiments did not allow us to examine the contribution of glucose clearance by peripheral tissue and glucose production to the *in vivo* response to glucoprivation, however, we measured counterregulatory hormones to determine whether altered glycemic response in *Bad* ^−/−^ mice might be related to differences in neurohumoral responses. Serum concentrations of counterregulatory hormones glucagon, epinephrine and corticosterone were measured at 30 minutes following 2DG administration in light of published reports that this time point elicits the strongest glucagon response [14]. Importantly, to discriminate between impaired glucoprivic responses and generalized stress responses, mice were anesthetized 30 minutes following saline or 2DG administration and blood samples collected. This experimental setup enabled us to distinguish between hormonal responses that result from general stress associated with animal handling, i.p. injections, and anesthesia that will be apparent in saline-treated animals, and the hormonal changes that are specific to glucoprivation appearing only with 2DG administration. In wild type mice, 2DG triggered a significant rise in plasma glucagon and epinephrine levels while in *Bad* ^−/−^ mice, these hormonal responses to glucoprivation were significantly blunted (Fig. 1B, C). Notably, serum epinephrine levels were comparable in saline-treated *Bad ^+/+^* and *Bad* ^−/−^ mice that were similarly anesthetized prior to blood collection (saline arm, Fig. 1C). These results suggest that alterations in 2DG-induced hormonal responses in BAD-deficient mice stem from changes in counterregulatory responses and cannot be explained by global alterations in catecholamine levels, which would be predicted in response to generalized stress such as anesthesia.

Glucagon and epinephrine responses are the foremost line of defense against glucoprivation. If glucose deficit persists, corticosterone release is triggered to compliment glucagon and epinephrine. 150 mg/kg 2DG induces a modest glucoprivic threat [14] that is not sufficient to induce corticosterone response in control mice (Fig. 1D). However, in *Bad* ^−/−^ mice, this level of glucoprivation mounted a significant corticosterone response (Fig. 1D). This is consistent with impaired glucagon and epinephrine responses in these mice, transforming the signal from 150 mg/kg 2DG to a greater glucoprivic threat. These data are also corroborated by lower glucose levels under these conditions (Fig. 1A). Thus, alterations in counterregulatory hormones in BAD-deficient mice parallel changes in glycemic profiles in glucoprivation studies. These observations suggest a role for BAD in proper detection of hypoglycemia and underscore its relevance for mediating hormonal responses to systemic glucoprivation.

### Comparable Glucagon Secretion in Primary *Bad ^+/+^* and *Bad* ^−/−^ Islet Cultures

**Figure 2:**
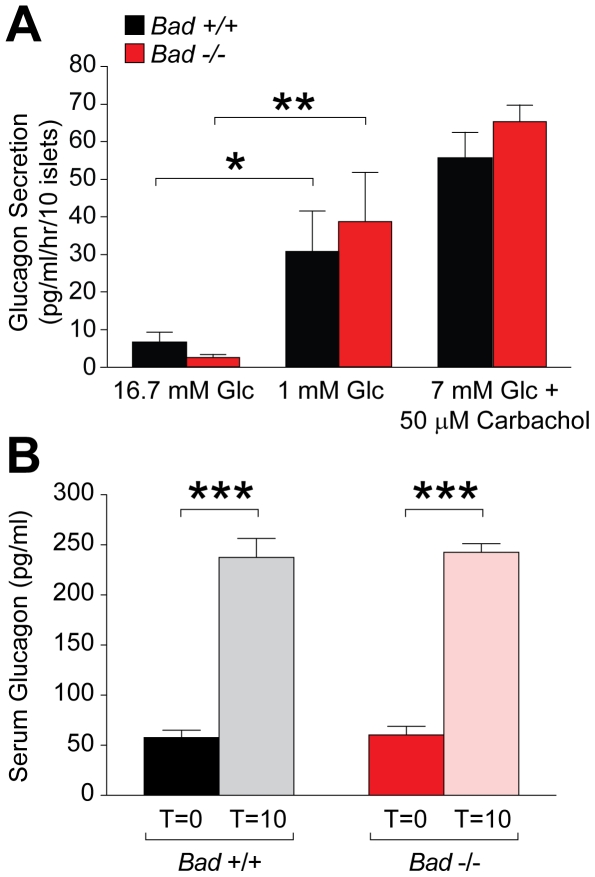
Glucagon secretion in response to glucose and arginine. (A) Glucagon secretion in primary *Bad* ^−/−^ and *Bad* ^+/+^ islets. Data are mean ± s.e.m and represent cumulative values from 3 independent islet isolations. Glc, glucose. *p<0.05; **p<0.01, unpaired two tailed *t*-test (B) Glucagon secretion during L-arginine stimulation of *Bad* ^−/−^ and *Bad* ^+/+^ mice (n = 4–7 per group). ***p<0.001, unpaired two tailed *t*-test.

Hypoglycemia-induced glucagon secretion is regulated by both autonomic input from the brain as well as direct glucose sensing by pancreatic islets [14], [16], [43], [44], [45]. As *Bad* ^−/−^ mice are a genetic model of total body BAD deficiency, the impaired glucagon response to glucoprivation could stem from defects in pancreatic or extrapancreatic glucose sensors. To determine the potential contribution of altered α-cell function to impaired glucagon response, the glucagon secretory capacity of primary *Bad ^+/+^* and *Bad* ^−/−^ islets in response to lowering glucose concentrations from high (16.7 mM) to low (1 mM) levels was assessed [18]. BAD-deficient islets displayed comparable glucagon secretory response to that observed in control islets (Fig. 2A). These observations suggest that the impaired glucagon response to glucoprivation in *Bad* ^−/−^ mice is less likely due to a cell-intrinsic defect in α-cells. Furthermore, glucagon response to the synthetic choline ester carbachol was comparable in *Bad* ^−/−^ and *Bad ^+/+^* islets (Fig. 2A). Thus, both the α-cell glucose sensing properties and its response to autonomic input are unaltered in *Bad* ^−/−^ mice.

In addition to hypoglycemia, certain amino acids, in particular L-arginine stimulate glucagon secretion [46]. Glucagon secretion in response to intraperitoneal administration of L-arginine was subsequently examined to determine whether impaired glucagon release in *Bad* ^−/−^ mice is specific for glucose-related signals or more general to nutrient-derived stimuli. Serum glucagon levels were measured at 10 minutes following arginine stimulation as this time point has been previously shown to display the strongest glucagon response [47]. L-Arginine-induced glucagon secretion was similar in *Bad ^+/+^* and *Bad* ^−/−^ mice (Fig. 2B), indicating that the nutrient sensing defect and the attendant impairment of glucagon response in the absence of BAD are glucose-selective.

### BAD-Dependent Counterregulatory Responses to Insulin-Induced Hypoglycemia

**Figure 3:**
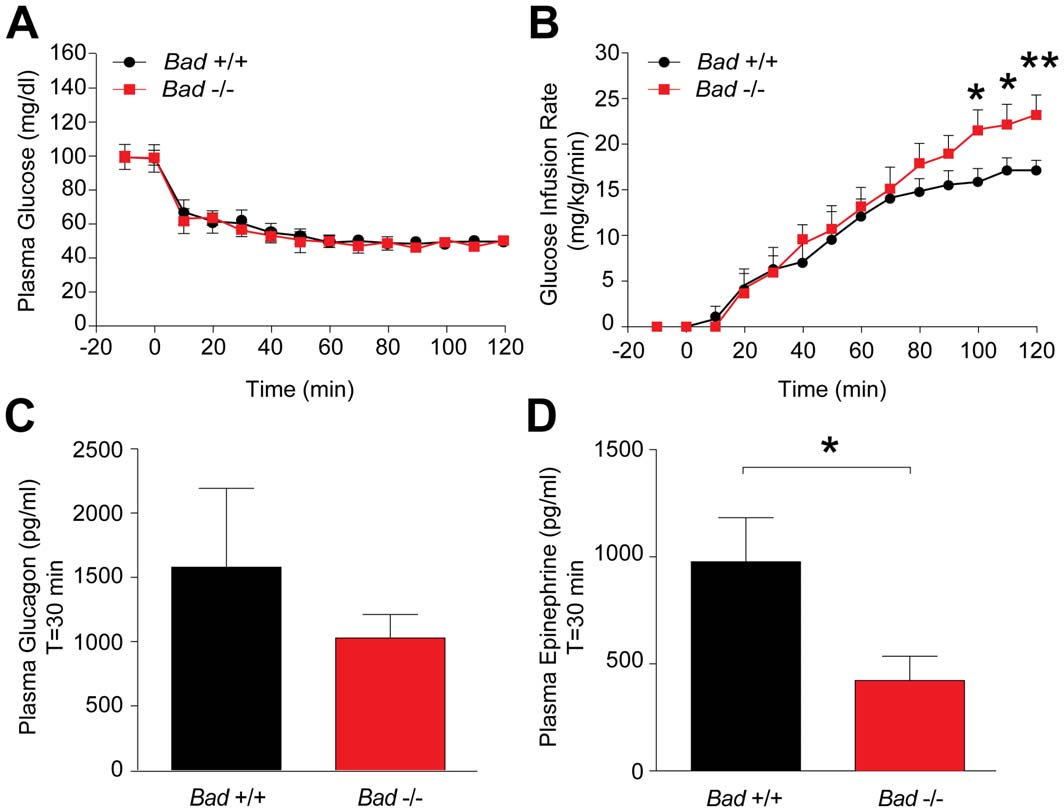
Impaired counterregulatory responses to insulin-induced hypoglycemia in BAD-deficient mice. Plasma glucose levels (A) and glucose infusion rate (GIR) (B) in *Bad* ^−/−^ and *Bad* ^+/+^ mice subjected to hyperinsulinemic hypoglycemic clamp analysis. Plasma glucagon (C) and epinephrine (D) levels at 30 min during the clamp period. *p<0.05; **p<0.01, *Bad* ^−/−^ vs *Bad* ^+/+^ mice (n = 9 per group), unpaired two tailed *t*-test.

While 2DG-induced glucoprivation is a useful experimental model of hypoglycemia, it does not precisely mimic the clinically relevant stimulus experienced by diabetic patients; namely insulin-induced hypoglycemia. To assess the role of BAD in the control of counterregulatory responses to insulin-induced hypoglycemia, a hyperinsulinemic hypoglycemic clamp approach was pursued. Plasma glucose levels indicated comparable hypoglycemia was achieved and maintained in *Bad* ^−/−^ and *Bad ^+/+^* mice throughout the analysis (Fig. 3A).

Within the last 30 minutes of the clamp period, glucose infusion rate (GIR) required to maintain hypoglycemia was significantly higher in *Bad* ^−/−^ mice (Fig. 3B). Differences in GIR may stem from changes in endogenous glucose output and/or peripheral glucose uptake. Examination of glucose tracers incorporated in the clamp study indicated comparable glucose turnover rates in *Bad* ^−/−^and *Bad ^+/+^* mice at both baseline (average turnover −10 to 0 min, 25±3 vs 24±4 mg/kg/min, respectively) and at the end of the clamp period (average turnover 80–120 min, 21±1 vs 21±1 mg/kg/min, respectively). As expected, endogenous glucose production was suppressed by the high dose of insulin during these studies, however, *Bad* ^−/−^ mice showed greater suppression (80–120 min, 1±2 vs 5±1 mg/kg/min, p = 0.05, *Bad* ^−/−^ vs *Bad ^+/+^*, respectively). Thus, the higher GIR in *Bad* ^−/−^ mice during the last 30 minutes of the clamp period may coincide with greater suppression of endogenous glucose output. These results are consistent with the idea that BAD deficiency is associated with counterregulatory deficit.

At 30 minutes during hypoglycemic clamp, plasma glucagon concentrations showed a lower trend *Bad* ^−/−^ mice (Fig. 3C). In these studies, glucagon responses to hypoglycemia in both genotypes were generally higher than typically reported in similar studies in mice. However, marked differences both between and within outbred mouse strains in responses to hypoglycemia have been reported [48], indicating the importance of appropriate littermate controls in these mouse clamp studies. Epinephrine levels at 30 minutes during hypoglycemic clamp were also measured and found to be significantly blunted in *Bad* ^−/−^ mice (Fig 3D). Of note, basal levels of glucagon and epinephrine were comparable in both genotypes with glucagon at 78±12 and 130±58 pg/ml and epinephrine at 41±19 and 43±21 pg/ml (*Bad* ^−/−^ vs *Bad ^+/+^*, mice, p>0.05).

Overall, these results indicate BAD deficiency is associated with impaired counterregulatory responses to insulin-induced hypoglycemia, a clinically relevant glucoprivic threat. The counterregulatory deficit in *Bad* ^−/−^ mice might be associated with greater suppression of endogenous glucose production in response to high dose of insulin in the experimental setting used for hyperinsulinemic hypoglycemic clamp studies. In addition, the possible contribution of reduced sympathetic tone as suggested by lower epinephrine levels in *Bad* ^−/−^ mice and the associated changes in peripheral insulin sensitivity to altered counterregulatory responses in these animals cannot be excluded.

### Impaired Counterregulatory Responses to Glucoprivation Following Acute Central Knockdown of *Bad*

**Figure 4:**
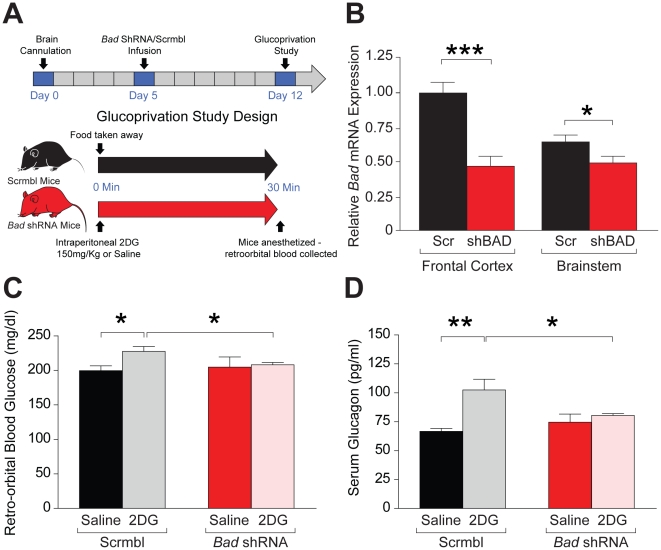
Impaired counterregulatory responses to glucoprivation following acute central knockdown of *Bad*. (A) Schematic representation of the experimental design. (B) Relative *Bad* mRNA levels in the frontal cortex and the brainstem (as representative fore- and hindbrain areas, respectively) 7 days after brain infusion of adenoviruses bearing *Bad* shRNA or control scrambled (scr or scrmbl) sequence, n = 7–9 mice per group. (C, D) Glycemic (C) and glucagon (D) responses to 2DG-induced glucoprivation in mice after stereotactic delivery of adenoviruses as in (A), n = 5–6 (saline) and n = 12–13 (2DG). Glucose and hormone values were assayed 30 min after 2DG (150 mg/kg) administration. *p<0.05; **p<0.01; ***p<0.001, unpaired two tailed *t*-test.

To determine whether abnormalities in hypoglycemia counterregulation in *Bad* ^−/−^ mice are directly due to central BAD deficiency *per se* or to potential secondary effects of total body BAD deficiency, the functional outcome of acute central *Bad* ablation was investigated (Fig. 4A). This genetic approach also helped determine whether BAD-dependent modification of brain glucose sensing as compared with pancreatic or extrapancreatic glucose sensors such as the portal vein is sufficient to phenocopy the defects in counterregulatory responses to glucoprivation observed in BAD-deficient mice.

Stereotactic delivery of adenoviruses bearing shRNA against *Bad* to C57/BL6J wild type mice effectively diminished its expression in the brain compared with a scrambled control sequence (Fig. 4B). Subsequent analysis of glycemic and glucagon responses to systemic glucoprivic challenge indicated that acute diminution of *Bad* expression in the brain is sufficient to impair hypoglycemia counterregulation. In response to intraperitoneal administration of 150 mg/kg 2DG, mice with brain knockdown of *Bad* failed to mount a glycemic response (Fig. 4C). Of note, the range of glucose values obtained using retro-orbital blood samples in these studies were significantly lower than tail blood glucose levels (Fig. 4C vs 1A). This is consistent with previous reports indicating that collection sites influence blood glucose concentration range [49]. Nonetheless, both BAD-deficient mice and animals with brain *Bad* knockdown had impaired glycemic response to glucoprivation compared with control cohorts independent of blood collection methods. Diminished expression of *Bad* in the brain was also linked to impaired glucoprivic stimulation of glucagon release (Fig. 4D). Importantly, glucagon levels were comparable in *Bad* shRNA and control cohorts administered with saline and similarly handled and treated for blood collection (saline arm, Fig. 4D), indicating that BAD-dependent changes in glucagon are selective to glucoprivic responses rather than an outcome of generalized stress responses. Taken together, these findings indicate that brain-specific acute knockdown of *Bad* is sufficient to impair detection of hypoglycemia and initiation of hormonal counterregulatory responses and highlight a role for central expression and function of BAD in brain glucose sensing.

### Regulation of Glucoprivic Feeding by BAD

**Figure 5:**
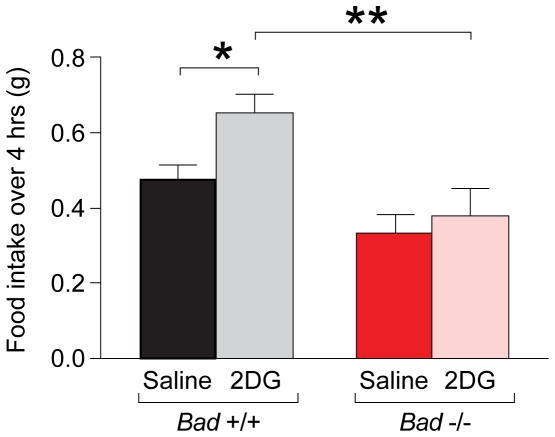
Genetic requirement of BAD in the glucoprivic feeding response. Glucoprivic feeding response 4 hrs after i.p. administration of 2DG (150 mg/kg) or saline to *Bad* ^−/−^and *Bad* ^+/+^ mice (n = 11–12 per group). *p<0.05; **p<0.01, unpaired two tailed *t*-test.

In addition to hormone release, glucoprivation triggers important symptomatic responses, including hunger, which is arguably the most important individual symptom of hypoglycemia [1]. Several lines of evidence suggest that the control of food intake is predominantly driven by the central nervous system [50], [51]. The brain integrates a myriad of signals [52], [53], including glucose availability to regulate food intake [54]. To determine the extent to which adaptive responses to hypoglycemia were altered by the absence of BAD, protective feeding responses to glucoprivation were also examined. Basal cumulative food intake over a 24 hour period was comparable in *Bad* ^−/−^ and *Bad ^+/+^* mice (data not shown), indicating that loss of BAD does not globally alter feeding behavior. However, basal light cycle food intake during the 4 hour period of 2DG-induced glucoprivation was significantly lower in *Bad* ^−/−^ mice compared with wild type controls (Fig. 5). Thus, changes in feeding behavior in face of BAD deficiency selectively manifest within the context of glucoprivation. These data indicate that the failure to mount proper responses to hypoglycemia in the absence of BAD extends beyond neurohumoral responses to effector functions such as glucoprivic feeding that are also regulated by central glucose sensing.

## Discussion

Here, we provide two independent lines of genetic evidence using *Bad*-null mice and shRNA-mediated whole brain knockdown of *Bad* in wild type mice that uncover a previously unappreciated role for this molecule in the control of hypoglycemia counterregulation. The relevance of BAD in this adaptive response is apparent in two different models of systemic hypoglycemia; 2DG-induced glucoprivation and the more clinically relevant paradigm of insulin-induced hypoglycemia. Our findings suggest that several functional effectors of central glucose sensing are modified by BAD, including neurohumoral responses culminating in secretion of glucagon and epinephrine, as well as glucoprivic feeding. The finding that brain knockdown of *Bad* impairs glycemic and glucagon responses to glucoprivation is consistent with the idea that BAD expression and function in the brain is required for proper hypoglycemia counterregulation and suggests the involvement of central gluco-detection. However, the precise neuroanatomical region(s) mediating BAD's effect in central glucose sensing and the underlying neurocircuitry remain to be determined. Importantly, our studies do not exclude potential involvement of other glucose sensors outside the brain such as those reported in the portal vein.

The initial detection of defective hypoglycemia counterregulation in BAD-deficient mice raised important questions regarding the contribution of non-glycemic and generalized stress responses versus glucose-specific changes in neuroendocrine responses. Two independent lines of observations suggest that the hormonal changes in these mice are less likely caused by non-glycemic stress. First, the epinephrine response to anesthetic stress is unaffected in *Bad* ^−/−^ mice (saline administered *Bad ^+/+^* vs *Bad* ^−/−^ mice, Fig. 1C). Second, the glucagon secretory response to L-arginine stimulation is preserved in these animals (Fig. 2B), congruent with the idea that BAD-dependent changes in glucagon responses are specific for glucose-related stimuli. 

In light of our previous findings that direct binding and activation of GK by BAD regulates glucose sensing and insulin secretion in β-cells [37], an initial prediction was that loss of BAD would phenocopy the neuroendocrine alterations associated with pharmacologic or genetic interference with GK [19], [21], [22]. Surprisingly, the main functional effector of central glucose sensing that appears to phenocopy loss-of-GK function in BAD-deficient mice is impaired glucoprivic feeding [22]. As for neurohumoral responses to insulin-induced hypoglycemia, diminution of GK [21] and loss of BAD have divergent effects. This suggests that BAD's role in the control of hormonal responses to hypoglycemia may be independent of GK. Several possibilities may explain the divergent effect of BAD and GK in this setting. For example, BAD has a much wider expression pattern in the brain compared with GK [38], [55], [56]. It is also possible that the neural mechanisms and the predominant neuroanatomical regions and glucose sensors in charge of glucoprivic feeding behavior versus hormonal responses are not exactly identical or redundant. This idea is consistent with other lines of evidence that have suggested separate mechanisms may control feeding versus hormonal responses [9]. Consequently, BAD-regulation of glucoprivic feeding may involve its partnership with GK, while its effects on hormonal responses may be mediated by other mechanisms. Furthermore, recent findings suggest that BAD may regulate certain aspects of neuronal functions particularly NMDA receptor-dependent synaptic transmission [57]. This may be relevant within the context of neurohumoral responses during hypoglycemia as central glutaminergic signaling *via* NMDA receptors controls neuroendocrine secretion [58], including counterregulatory responses to hypoglycemia [30], [31]. Lastly, BAD-dependent modulation of calcium buffering [59] may further shape the nature of calcium signals in these neurochemical responses. Future studies and new genetic tools are required to test each of the aforementioned possibilities and delineate the predominant mechanism(s) underpinning BAD's role in hypoglycemia counterregulation. Further dissection of BAD as a molecular modulator of brain glucose sensing and its potential functional collaboration with other known glucose sensors may uncover novel insights as well as new therapeutic targets for the control of hypoglycemia caused by aggressive insulin therapy.
